# Supplementary material for: Neural processing of working memory in adults with ADHD in a visuospatial change detection task with distractors
Source: PeerJ. 2018 Sep 18;6:e5601. doi: 10.7717/peerj.5601 (PMC6149497; doi:10.7717/peerj.5601)
Supplement: Supplemental Information 1 [file peerj-06-5601-s001.docx]

SUPPLEMENTARY INFORMATION

Sample selection

The present study was part of a larger project investigating the neural correlates of working memory training in college students with ADHD. This larger project consisted of several phases during its execution. The participants we used for the present study were taken from what we called the ‘pilot-Engage’ phase (see Mawjee et al., 2014) and the ‘Engage’ phase (see Mawjee et al., 2015). Across both phases, the Change Detection task described as well as the other outcome measures in the present manuscript were identical.

We report this information in case readers wish to make comparisons between different past (and future) manuscripts within the larger project to, for example, avoid the duplicate reporting of findings from our samples in meta-analytical studies. The differences between the ‘pilot-Engage’ and ‘Engage’ sample pertained to training-related procedures the participants with ADHD underwent *after* their first visit. The pilot-Engage sample was intended to optimize procedures, make potential changes to, and estimate sample sizes for, the larger Engage sample. Differences between samples were minor and consisted of improved standardization of the coach calls for the waitlist group, the recommended use of a planner to reduce study attrition, and the addition of post-training interviews (see also, Mawjee et al. (2014), for details).

The ADHD group consisted of 32 participants in the pilot-Engage and 103 participants in the Engage Group for a total of 135 participants. After data cleaning, 125 participants had usable EEG data. Twenty-seven individuals in the Comparison group individuals were collected continuously during both these phases and all had usable data. When funding for the project began to run low, we prioritized ADHD sample which underwent training at the expense of a large comparison group explaining the discrepancy in numbers.

ADHD subjects were matched to the 27 subjects in the comparison group. ADHD subjects were pseudo-randomly selected on the criteria of gender, IQ, age, and medication status. Forty-nine ADHD subjects were eligible to be matched of which 27 were selected. To address any bias in the randomized assignment of the sample selection, we compared the remaining 22 unmatched eligible ADHD sample with the matched ADHD sample on the ASRS, CFQ, and Digit Span. The analyses showed that these groups were not statistically different (all *p*’s > .55).

**Figure S1:** Electrode site selection for CDA using the 129 channel Geodesic net configuration.


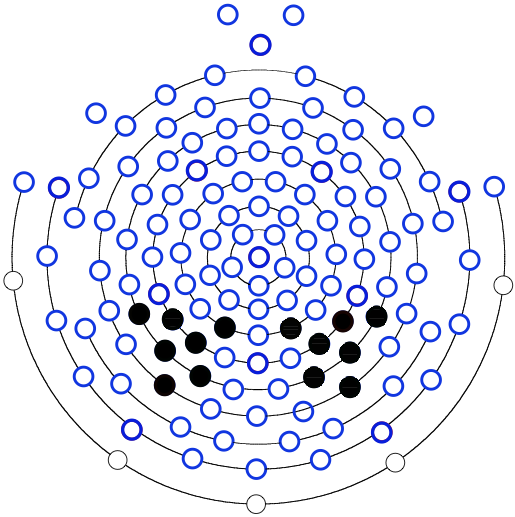


References:

Mawjee, K., Woltering, S., Lai, N., Gotlieb, H., Kronitz, R., & Tannock, R. (2014). Working memory training in ADHD: controlling for engagement, motivation, and expectancy of improvement (pilot study). *Journal of attention disorders*, 1087054714557356.

Mawjee, K., Woltering, S., & Tannock, R. (2015). Working memory training in post-secondary students with ADHD: A randomized controlled study. *PloS one*, *10*(9), e0137173.
